# Effect of Partial or Complete Substitution of Fish Meal by Meat Meal in the Feed of Red Sea Bream (*Pagrus major*) on the Growth Performance and Feed Utilization

**DOI:** 10.1155/anu/9589317

**Published:** 2025-03-27

**Authors:** Yu Jin Sim, Sung Hwoan Cho

**Affiliations:** ^1^Department of Convergence Interdisciplinary Education of Maritime and Ocean Contents, Korea Maritime and Ocean University, Busan 49112, Republic of Korea; ^2^Division of Convergence on Marine Science, Korea Maritime and Ocean University, Busan 49112, Republic of Korea

**Keywords:** feed availability, fish meal replacer, growth, meat meal, *Pagrus major*

## Abstract

As fish meal (FM) has become an expensive protein source to sustainably use in aquafeeds due to its stagnant production and increased price, finding a FM replacer is essential for the advancement of sustainable aquaculture. This study examined the effect of partial or complete FM substitution by meat meal (MM) in feeds on the growth and feed utilization of red sea bream (*P. major*). Six diets with isoproteic and isolipidic were prepared. In the control (Con) diet, 55% FM was included. The partial (20%, 40%, 60%, and 80%) or complete (100%) of FM levels in the Con diet were substituted by MM, named the MM20, MM40, MM60, MM80, and MM100 diets, respectively. Four hundred and fifty juvenile (7.85 ± 1.851 g; mean ± SE) red sea bream were distributed into 18 tanks (25 juvenile/tank) with triplicate. Throughout an 8-week feeding period, all fish were carefully hand-fed twice daily. At the completion of the 8-week experimental period, weight gain, specific growth rate, and feed consumption of fish fed the Con, MM20, and MM40 diets were superior to fish fed the MM60, MM80, and MM100 diets. Feed efficiency of fish fed the MM20 diet was superior to fish fed the MM100 diet, but comparable to fish fed the Con, MM40, MM60, and MM80 diets. Protein efficiency ratio of fish fed the Con, MM20, MM40, and MM60 diets was higher than that of fish fed the MM100 diet. Protein retention of fish fed the Con, MM20, and MM40 diets was superior to fish fed the MM100 diet. The condition factor of fish fed the Con diet was higher than that of fish fed the MM80 and MM100 diets. Plasma and serum parameters and biochemical composition of fish, except for fatty acid profiles were not significantly influenced by dietary MM supplementation as a substitution for FM. In conclusion, FM up to 40% could be substitutable by MM in a 55% FM-based feed without significantly impairing the growth performance and feed consumption of red sea bream.

## 1. Introduction

Aquaculture technology has rapidly grown to provide a great source of protein and various nutrients including minerals and vitamins [1]. Approximately 68% of fish and crustacean farms use commercially formulated fish meal (FM)-basal diet [2]. FM has been commonly used as a main protein in aquafeeds due to its balanced of amino acid (AA) and fatty acid (FA) profiles, abundant minerals and vitamins, and high palatability [3]. In general, carnivorous fish including red sea bream (*Pagrus major*) require dietary FM over 40% [3–5] for optimal growth. However, the global FM production has plateaued or decreased because of climate change and its global price is rising due to elevated demand for FM resulting from the development of aquaculture worldwide [6]. Therefore, scientists need to find the cost-effective and nutritionally balanced protein ingredients as a FM replacer for the advancement of sustainable aquaculture.

Many studies have reported on various terrestrial animal (meat meal [MM] [7], meat and bone meal [MBM] [4], porcine blood meal [5], and poultry by-product meal [PBM] [8]), aquatic animal (marine fish processing by-products [9], and shrimp head meal and viscera meal from pen shell and catarina scallop [10]), and plant (corn gluten meal [CGM] [11], cottonseed protein concentrate [12], and soy protein concentrate [SPC] [13]) proteins as a FM replacer in fish feeds. However, issues of hygiene challenges due to the short shelf-life and high perishability of aquatic animal by-products [14], and the lower protein and essential AA (EAA) and higher carbohydrate and antinutritional factors in plant by-products compared to FM [15] still limited their use as a FM replacer in fish diets. Therefore, searching for terrestrial animal protein source as a FM substitute may provide a promising approach to improve the nutritional sustainability of fish feeds.

MM and MBM, which were types of terrestrial animal by-product meals, were obtained by removing lipids, heating, and compressing from inedible parts including meat scraps and internal organs of livestock, such as swine and cattle during the slaughter process [16]. However, high MBM replacement for FM in diets caused poor growth and high mortality of fish [17, 18] since MBM contains a high (26.9%–28.7%) ash content because of the presence of bone [4, 19]. Thus, the substitutability of FM by MBM in fish feeds seems to be very limited. On the other hand, MM, unlike MBM, has a relatively low ash content due to the absence of bone, enhancing the substitutability of FM, and its cost (USD 0.88/kg, USD 1 = 1300 KRW) is lower than FM (USD 2.17/kg), so MM as a FM alternative in fish feeds could lead to stable and sustainable aquaculture practices. MM is classified into low-quality and high-quality MM based on their protein content, and particularly, high-quality MM contains higher (≥80%) crude protein (CP) and lower (4.0%–8.7%) ash content [7, 20, 21] compared to low-quality MM with CP less than 75% and 12.6%–17.9% ash content [22–24]. High-quality MM also led to comparable or superior digestibility compared to FM in diets for juvenile silver perch (*Bidyanus bidyanus*) [25] and juvenile and grower rockfish (*Sebastes schlegeli*) [26]. Furthermore, the substitutability of FM with MM could be profoundly influenced by the quality of MM. For instance, FM up to 30% could be replaceable by low-quality (CP: 70.5%) porcine MM in a 30% FM-basal diet without worsening growth and feed efficiency (FE) of golden pompano (*Trachinotus ovatus*) [27]. However, high-quality MM (CP: 80.3%) could substitute FM up to 40% in a 65% FM-basal diet without declining growth and feed availability of olive flounder (*Paralichthys olivaceus*) [7]. In contrast, high-quality MM (CP: 80%) supplemented with AA could replace FM up to 60% in an 80% FM-based diet without compromising the growth of olive flounder [21]. Given these results, high-quality MM can also be a promising FM alternative in the diet of red sea bream, offering both nutritional and economic benefits.

Red sea bream, a carnivorous fish species, is a valuable species of marine fish in Eastern Asia, especially in Japan and Republic of Korea (*hereafter*, Korea), and its farming began in Korea in the 1980s [28]. The production (6283 metric tons) of this species accounted for ~8% of total aquaculture production (79,651 metric tons), and its value was reported to be USD 10.8 per kg of fish in Korea in 2023 [29], indicating that it is a high-value fish species. Accordingly, a variety of studies on the substitutability of animal and plant sources for FM in the feeds of red sea bream have been conducted [5, 30–33]. In particular, Gunathilaka et al. [20] proved that 50% of FM could be replaceable with high-quality MM (CP: 84.2%) without compromising growth, feed consumption, and FE when fingerling (mean initial weight of 50.2 g) red sea bream were provided with a 60% FM-basal diet or one of diets replacing 50% of FM with plants (CGM and SPC), animals (chicken by-product meal and high-quality MM), or their blends for 12 weeks.

The size (age) of fish can greatly influence the substitutability of FM by alternative proteins in diets. In general, smaller (younger) fish are less likely to tolerate higher level of FM substitution [34, 35]. For example, Takagi et al. [36] proved that FM up to 30% and 70% could be substitutable with CGM in the 50% FM-based feeds of fingerling (mean initial weight of 53 g) and yearling (mean initial weight of 280 g) red sea bream, respectively, without compromising growth performance in the 40- and 232-day feeding experiments, respectively. However, no study on the substitutability of MM for fish in the juvenile red sea bream has been performance yet.

This experiment, thus, aims to estimate the impacts of partial or complete FM substitution by high-quality MM in the diet of juvenile red sea bream on the growth, feed consumption, and feed utilization.

## 2. Materials and Methods

### 2.1. Arrangement of Red Sea Bream and Rearing Condition

Juvenile fish of similar sizes were bought from a hatchery (Tongyeong-si, Gyeongsangnam-do, Korea) and acclimatized to the rearing conditions for 2 weeks. Throughout the 2-week acclimatization period, red sea bream was supplied with a commercial extruded pellet (Suhyup Feed, Gyeongsangnam-do, Korea) including 50% CP and 13% crude lipid (CL) twice a day at 1.5%–3.0% of the total biomass. After the acclimatization period, 450 juvenile (7.85 ± 1.851 g; mean ± SE) red sea bream were randomly divided into 18 of 50-L rectangle flow-through tanks (water volume: 40 L) with triplicate (25 fish/tank). The water supplied to each tank was the blend of underground seawater and sand-filtered seawater in an equal proportion, and the flow rate of each tank was 4.4 L/min. Water quality was monitored using a digital multimeter (AZ-8603; AZ Instrument Corp., Taichung, Taiwan) daily after feeding in the morning. The water temperature altered from 17.7 to 23.2°C (20.7 ± 1.48°C; mean ± SD), dissolved oxygen altered from 7.4 to 8.3 mg/L (7.7 ± 0.27 mg/L), salinity altered from 29.7 to 31.5 g/L (30.5 ± 0.40 g/L), and pH altered from 7.3 to 7.7 (7.5 ± 0.07). The bottom of each tank was siphon-cleaned daily, and dead fish were promptly removed on observation. The light cycle mirrored natural conditions.

### 2.2. Arrangement of the Experimental Feeds

Six isoproteic (51.5%) and isolipidic (14.5%) feeds were prepared to meet the nutritional demands of red sea bream [37] (Table 1). To achieve CP of 51.5% in the experimental diets, 55% FM was included in the control (Con) diet. Fifty-five percent FM and 17% fermented soybean meal, 17.5% wheat flour, and 4% each of fish oil and soybean oil were included as the protein, carbohydrate, and lipid sources, respectively, in the Con diet. The partial (20%, 40%, 60%, and 80%) and complete (100%) of FM levels in the Con diet were replaced by high-quality MM (CP: 83.9% and CL: 9.9%) based on Ha, Cho, and Kim [7] and Sato and Kikuchi [21], named the MM20, MM40, MM60, MM80, and MM100 diets, respectively.

All ingredients were fully blended with water at a ratio of 3:1 and pelletized using a pelletizing machine (SMC-32; SL Company, Incheon Metropolitan City, Korea) with a diameter of 3 mm. All experimental diets were dried using a machine for drying (JW-1350ED; Jinwoo Electronics Co., Ltd., Hwaseong-si, Gyeonggi-do, Korea) for 48 h at 45°C, and stored in a freezer at –20°C until use. The experimental feeds were hand-fed twice (08:30 and 17:30) a day for 8 weeks until red sea bream was apparently satiated. The amount of feed provided to each tank was recorded daily, but uneaten feed was not collected.

### 2.3. Growth Measurements of Fish

At the completion of the 8-week experimental period, all live fish were famished for 24 h, and then anesthetized using 100 mg/L tricaine methanesulfonate (MS-222). The live fish in each tank were counted and weighed collectively. Ten anesthetized fish from each tank were randomly chosen to determine condition factor (CF), viscerosomatic index (VSI), and hepatosomatic index (HSI). The following formulas were used to calculate growth, feed availability, and biological indices of red sea bream: specific growth rate (SGR, %/day) = (Ln final weight of fish [g] − ln initial weight of fish [g]) × 100/days of the feeding trial (56 days), FE = (total final weight of fish [g] − total initial weight of fish [g] + total weight of dead fish [g])/total feed consumption (g), protein efficiency ratio (PER) = weight gain of fish (g)/protein consumption (g), protein retention (PR, %) = protein gain of fish (g) × 100/protein consumption (g), CF (g/cm^3^) = body weight of fish (g) × 100/total length of fish (cm)^3^, VSI (%) = viscera weight of fish (g) × 100/body weight of fish (g), and HSI (%) = liver weight of fish (g) × 100/body weight of fish (g).

### 2.4. Plasma and Serum Measurements of Fish

Prior to blood samples collection for blood chemistry analysis, body weight of fish was assessed to estimate CF, and fish were dissected for VSI and HSI measurements after blood collection. Blood samples were obtained using heparinized syringes from the caudal veins of five anesthetized fish per tank. After centrifugation at 2700 × *g* at 4°C for 10 min, the plasma samples were acquired from the blood samples, and they were preserved in a deep freezer at –70°C until use. Alanine aminotransferase (ALT), aspartate aminotransferase (AST), alkaline phosphatase (ALP), total bilirubin (T-BIL), total cholesterol (T-CHO), triglycerides (TGs), total protein (TP), and albumin (ALB) of the plasma samples were assessed using an automatic dry-chemistry analyzer (FUJI DRI-CHEM NX500i; FUJIFILM Corp., Tokyo, Japan).

Blood samples were obtained using syringes from the caudal veins of five anesthetized fish per tank. After centrifugation at 2700 × *g* at 4°C for 10 min, the serum samples were obtained from the blood samples, and they were preserved in a deep freezer at –70°C until use. A superoxide dismutase (SOD) ELISA kit (MBS705758; MyBiosource Inc., San Diego, CA, USA) was utilized for SOD analysis. The analysis used a competitive inhibition enzyme immunoassay technique, in which the color development was inversely proportional to the SOD concentration in the samples. The concentration was determined by creating a standard curve. Absorbance was performed using a microplate reader (Infinite 200 PRO; Tecan Group Ltd., Männedorf, Canton of Zürich, Switzerland) at 450 nm.

Turbidity measurement was conducted for lysozyme activity assay according to Lange, Guđmundsdottir, and Magnadottir [38]. Briefly, 1.9 mL *Micrococcus lysodeikticus* (Sigma–Aldrich Inc., St Louis, MO, USA) was suspended in 0.05 M phosphate-buffered saline (pH 6.2). A total of 100 µL sample, consisting of 95 µL suspension and 5 µL of the serum sample, was incubated at 25°C for 5 min, and absorbance readings were performed at 530 nm at 0, 15, 45, and 60 min. The absorbance assay was performed using the same machine as the SOD assay. The enzyme amount needed to generate a 0.001/min drop in absorbance was used to measure lysozyme activity.

### 2.5. Biochemical Composition of the Experimental Diets and Whole-Body Fish

The main proteins (FM and MM), all experimental feeds, 10 juvenile fish before the feeding experiment, and all remaining (≥9) fish in each tank were homogenized for the proximate analysis. The proximate compositions of the samples were analyzed following AOAC standard method [39]. The moisture content of the dry and wet samples was analyzed using a drying oven at 105°C for 6 and 24 h, respectively. The CP and CL content were assessed using the Kjeldahl method (Kjeltec 2100 Distillation Unit, Foss, Hillerød, Denmark) and ether-extraction method (Soxtec 2043 Fat Extraction System, Foss, Hillerød, Denmark), respectively. The ash content was assessed using a muffle furnace for 4 h at 550°C. The gross energy of all experimental diets was measured using a bomb calorimeter (Model 6100; Parr Instrument Co., Moline, IL, USA).

The AA profiles, except for tryptophan in the samples were evaluated using an L-8800 auto-analyzer (Hitachi, Tokyo, Japan). This analysis was performed after fish were hydrolyzed with 6 N HCl at 110°C for 24 h. The tryptophan analysis was performed using a S1125 HPLC pump system (Sykam GmbH, Eresing, Germany).

The FA profiles were checked by comparing the sample to a known standard, 37-component FAME mix CRM47885 (Supelco, St. Louis, MO, USA). According to Folch, Lees, and Stanley [40], lipids for the analysis of FA were extracted from the samples using a combination of chloroform and methanol at a ratio of 2:1. The extracted lipids were then methylated using 14% BF3-MeOH, and analyzed using a gas chromatograph (HP 6890; Agilent Technologies Inc., Santa Clara, CA, USA) fitted with an SP-2560 capillary column (100 m × 0.25 mm inner diameter and 0.20 μm film thickness; Supelco, St. Louis, MO, USA). FA peaks were identified by comparing them to the methyl esters of standard FA.

### 2.6. Statistical Analysis

All means of parameters were evaluated by the normality test and homogeneity of variances test (Shapiro–Wilk and Levene's test, respectively). Statistical differences among the means were estimated by one-way ANOVA and Tukey's honestly significant difference (HSD) test by using IBM SPSS Statistics for Windows, version 24.0 (IBM Corp., Armonk, NY, USA). A *p*-value less than 0.05 was regarded as statistical differences. Prior to conducting statistical analysis, the percentage data were arcsine-transformed. The analysis of orthogonal polynomial contrasts was evaluated to decide whether statistical differences were linear, quadratic, or cubic. For statistical differences data, regression analysis was performed to fit a suitable model. The *p*-value was used to assess a proper dietary FM replacement level with MM for the dependent variable. Principal component analysis (PCA) was performed using SPSS version 24.0 to determine major contributing factors or patterns among the FA profiles of the whole-body fish. Additionally, to analyze the relationship between the FA profiles of the whole-body fish and dietary treatments, average linkage hierarchical clustering was conducted using Pearson correlation after log_2_ transformation of the data.

## 3. Results

### 3.1. AA Profiles of the Main Protein Sources (FM and MM) and Experimental Diets

**Figure 1:**
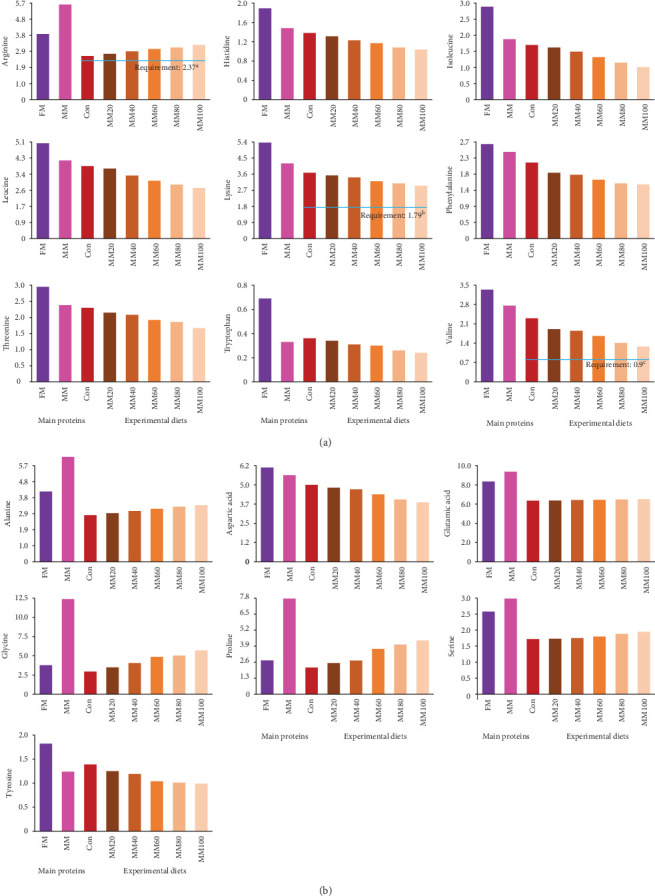
Amino acid (% of the diet) profiles of the main proteins and the experimental diets. (A) Essential amino acids. (B) Nonessential amino acids. FM, fish meal; MM, meat meal; Con, 55% FM-based diet; MM20, dietary 20% FM substitution with MM; MM40, dietary 40% FM substitution with MM; MM60, dietary 60% FM substitution with MM; MM80, dietary 80% FM substitution with MM; and MM100, dietary 100% FM substitution with MM. ^a^Arginine, ^b^lysine, and ^c^valine requirements were obtained from Rahimnejad and Lee's [41], Forster and Ogata's [42], and Rahimnejad and Lee's [43] studies, respectively.

All EAA, except for arginine, and aspartic acid and tyrosine among nonessential AA (NEAA) in FM were comparatively high over those in MM (Figure 1A,B, respectively). Arginine increased, but the rest of all other EAA decreased with dietary elevated FM replacement by MM, while aspartic acid and tyrosine decreased, but the rest of all other NEAA increased with dietary elevated FM substitution by MM.

### 3.2. FA Profiles of the Main Proteins and Experimental Feeds

**Figure 2:**
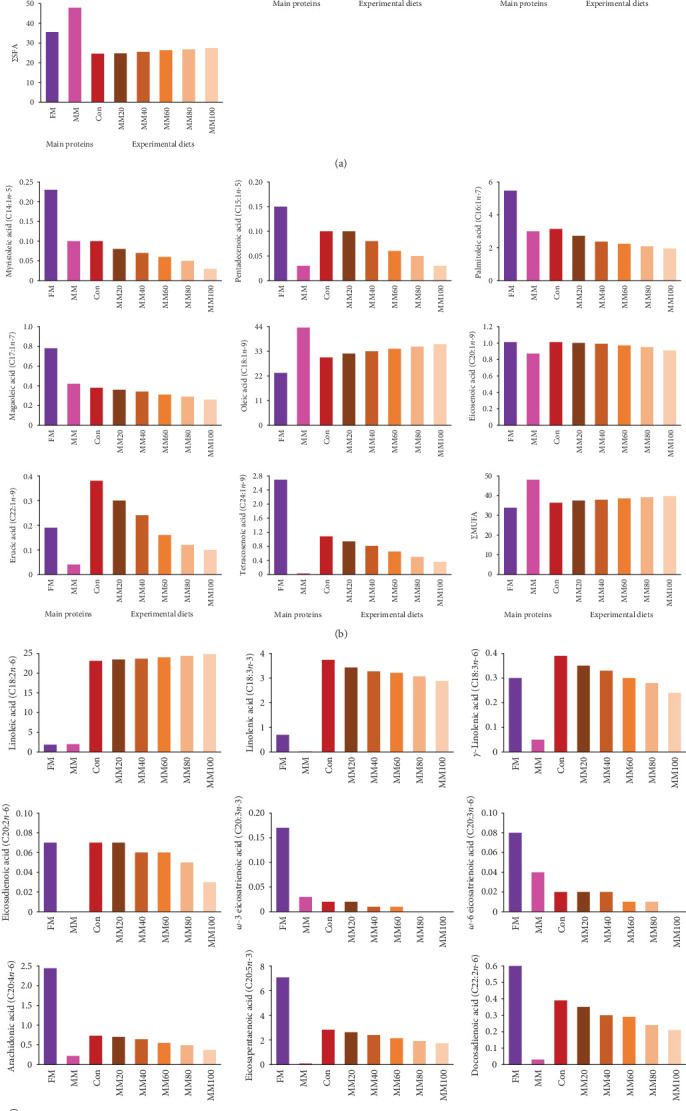
Fatty acid (% of total fatty acids) profiles of the main proteins and the experimental diets. (A) Saturated fatty acids. (B) Monounsaturated fatty acids. (C) Polyunsaturated fatty acids. FM, fish meal; MM, meat meal; Con, 55% FM-based diet; MM20, dietary 20% FM substitution with MM; MM40, dietary 40% FM substitution with MM; MM60, dietary 60% FM substitution with MM; MM80, dietary 80% FM substitution with MM; MM100, dietary 100% FM substitution with MM; ∑SFAs, total saturated fatty acids; ∑MUFAs, total monounsaturated fatty acids; ∑*n*-3 HUFAs, total *n*-3 highly unsaturated fatty acids; and DHA/EPA, the ratio of docosahexaenoic acid to eicosapentaenoic acid.

Total saturated FA (∑SFA) and monounsaturated FA (∑MUFA) in MM were comparatively high over those in FM, while total *n*-3 highly unsaturated FA (∑*n*-3 HUFA) in FM was comparatively high over that in MM (Figure 2AB, and –C, respectively). The ∑SFA and ∑MUFA increased, but the ∑*n*-3 HUFA decreased with dietary elevated MM substitution for FM.

### 3.3. Survival and Growth of Red Sea Bream

**Figure 3:**
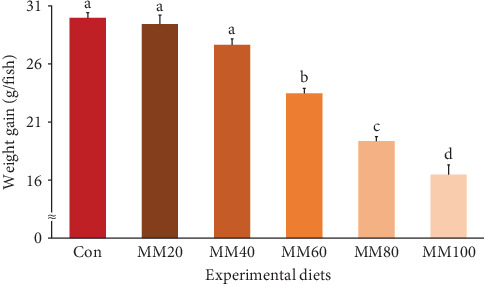
Weight gain (g/fish) (regression analysis; *Y* = – 0.145434*X* + 31.6540, *R*^2^ = 0.9253, *p* < 0.0001) (orthogonal polynomial contrast; linear: 0.000, quadratic: 0.002, cubic: 0.030) of red sea bream fed the experimental diets (means of triplicate ± SE) (*p* < 0.0001). Con, 55% fish meal (FM)–based diet; MM20, dietary 20% FM substitution with meat meal (MM); MM40, dietary 40% FM substitution with MM; MM60, dietary 60% FM substitution with MM; MM80, dietary 80% FM substitution with MM; and MM100, dietary 100% FM substitution with MM.

**Figure 4:**
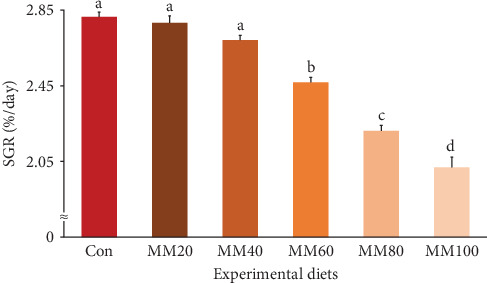
Specific growth rate (SGR, %/day) (regression analysis; *Y* = – 0.008442*X* + 2.9195, *R*^2^ = 0.9161, *p* < 0.0001) (orthogonal polynomial contrast; linear: 0.000, quadratic: 0.001, cubic: 0.073) of red sea bream fed the experimental diets (means of triplicate ± SE) (*p* < 0.0001). Con, 55% fish meal (FM)–based diet; MM20, dietary 20% FM substitution with meat meal (MM); MM40, dietary 40% FM substitution with MM; MM60, dietary 60% FM substitution with MM; MM80, dietary 80% FM substitution with MM; and MM100, dietary 100% FM substitution with MM.

Survival of fish ranged from 81.33% to 90.67%, but it was not statistically (*p* > 0.7) impacted by FM substitution with MM in diets (Table 2). Weight gain and SGR of fish fed the Con, MM20, and MM40 diets were superior (*p* < 0.0001 for all) to fish fed the MM60, MM80, and MM100 diets (Figures 3 and 4, respectively). In orthogonal polynomial contrast, weight gain of red sea bream revealed linear (*p* ≤ 0.001), quadratic (*p*=0.002), and cubic (*p*=0.030) relationships with dietary MM substitution levels for FM, but SGR of red sea bream revealed linear (*p* ≤ 0.001) and quadratic (*p*=0.001) relationships with dietary elevated MM replacement for FM. In regression analysis, linear relationships were revealed to be the most suitable model between dietary elevated MM substitution for FM (X) and weight gain (*Y* = – 0.145434*X* + 31.6540, *R*^2^ = 0.9253, *p* < 0.0001) and SGR of fish (*Y* = – 0.008442*X* + 2.9195, *R*^2^ = 0.9161, *p* < 0.0001), respectively.

### 3.4. Feed Consumption and Utilization, and Biological Indices of Red Sea Bream

**Figure 5:**
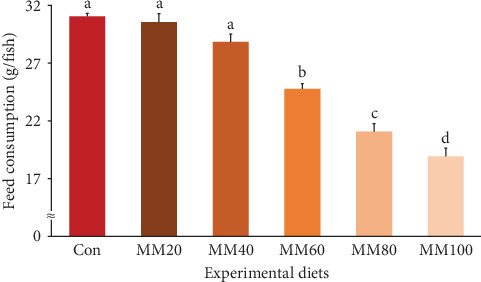
Feed consumption (g/fish) (regression analysis; *Y* = – 0.133145*X* + 32.5305, *R*^2^ = 0.9213, *p* < 0.0001) (orthogonal polynomial contrast; linear: 0.000, quadratic: 0.014, cubic: 0.019) of red sea bream fed the experimental diets (means of triplicate ± SE) (*p* < 0.0001). Con, 55% fish meal (FM)-based diet; MM20, dietary 20% FM substitution with meat meal (MM); MM40, dietary 40% FM substitution with MM; MM60, dietary 60% FM substitution with MM; MM80, dietary 80% FM substitution with MM; and MM100, dietary 100% FM substitution with MM.

Feed consumption of fish fed the Con, MM20, and MM40 diets was statistically (*p* < 0.0001) higher than that of fish fed the MM60, MM80, and MM100 diets (Figure 5). FE of fish fed the MM20 diet was statistically (*p* < 0.04) higher than that of fish fed the MM100 diet, but not statistically (*p*  > 0.05) different from that of fish fed the Con, MM40, MM60, and MM80 diets. PER of red sea bream fed the Con, MM20, MM40, and MM60 diets was statistically (*p* < 0.01) higher than that of fish fed the MM100 diet, but not statistically (*p*  > 0.05) different from that of fish fed the MM80 diet. PR of fish fed the Con, MM20, and MM40 diets was statistically (*p* < 0.006) higher than that of fish fed the MM100 diet, but not statistically (*p*  > 0.05) different from that of fish fed the MM60 and MM80 diets. In orthogonal polynomial contrast, feed consumption of red sea bream revealed linear (*p* ≤ 0.001), quadratic (*p*=0.014), and cubic (*p*=0.019) relationships with dietary increased MM replacement for FM, but FE, PER, and PR of fish revealed linear (*p*=0.002, *p*=0.001, and *p* ≤ 0.001, respectively) relationships with dietary elevated MM replacement for FM. In regression analysis, linear relationships were revealed to be the most suitable model between dietary replacement levels of FM by MM (X) and feed consumption (*Y* = –0.133145*X* + 32.5305, *R*^2^ = 0.9213, *p* < 0.0001) and PR (*Y* = –0.028218*X* + 31.4833, *R*^2^ = 0.6270, *p* < 0.0001), respectively. However, quadratic relationships were shown to be the most suitable model between dietary increased MM replacement for FM (X) and FE (*Y* = –0.00001051*X*^2^ + 0.000226*X* + 0.9763, *R*^2^ = 0.5908, *p* < 0.001) and PER (*Y* = –0.00002583*X*^2^ + 0.000987*X* + 1.8367, *R*^2^ = 0.6685, *p* < 0.0001), respectively, in regression analysis.

CF of fish fed the Con diet was statistically (*p* < 0.001) higher than that of fish fed the MM80 and MM100 diets, but not statistically (*p*  > 0.05) different from that of fish fed the MM20, MM40, and MM60 diets. In orthogonal polynomial contrast, CF of fish revealed linear (*p* ≤ 0.001) relationship with dietary FM replacement levels by MM. In regression analysis, linear relationship was revealed to be the most suitable model between dietary MM replacement for FM (X) and CF (*Y* = –0.003384*X* + 1.9533, *R*^2^ = 0.7451, *p* < 0.0001). VSI ranged from 8.61% to 8.73% and HSI ranged from 2.70% to 2.82%, but VSI and HSI were not statistically (*p* > 0.9 for both) influenced by dietary MM replacement for FM.

### 3.5. Blood Chemistry of Red Sea Bream

Plasma AST changed from 49.3 to 51.3 U/L, ALT changed from 7.3 to 7.7 U/L, ALP changed from 169.2 to 179.9 U/L, T-BIL changed from 0.3 to 0.4 mg/dL, T-CHO changed from 257.2 to 271.1 mg/dL, TG changed from 371.6 to 416.6 mg/dL, TP changed from 4.7 to 5.0 g/dL, and ALB changed from 1.0 to 1.2 g/dL (Table 3). Serum SOD changed from 3.8 to 4.2 ng/mL and lysozyme activity changed from 44.0 to 55.1 U/mL. None of the plasma and serum measurements were statistically (*p* > 0.05 for all) affected by dietary MM replacement levels for FM.

### 3.6. The Whole-Body Proximate Composition of Fish

The whole-body moisture content changed from 68.5% to 69.2%, CP changed from 16.4% to 16.8%, CL changed from 8.3% to 8.7%, and ash changed from 4.3% to 4.7% (Table 4). All experimental feeds did not statistically (*p* > 0.08, *p* > 0.05, *p* > 0.2, and *p* > 0.09, respectively) change the proximate composition of the whole-body fish.

### 3.7. The Whole-Body AA Profiles of Red Sea Bream

The AA profiles of the whole-body fish were not statistically (*p* > 0.05 for all) influenced by dietary MM substitution for FM (Table 5).

### 3.8. The Whole-Body FA Profiles of Fish

**Figure 6:**
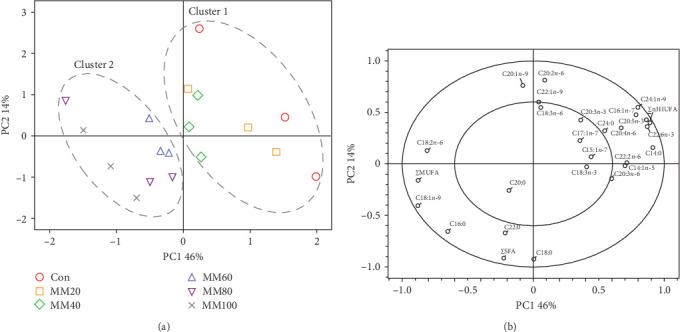
Principal component analysis (PCA) for the fatty acid profiles of the whole-body red sea bream fed the experimental diets: (A) score plot and (B) correlation loading plot. Con, 55% fish meal (FM)–based diet; MM20, dietary 20% FM substitution with meat meal (MM); MM40, dietary 40% FM substitution with MM; MM60, dietary 60% FM substitution with MM; MM80, dietary 80% FM substitution with MM; MM100, dietary 100% FM substitution with MM; ∑SFAs, total saturated fatty acids; ∑MUFAs, total monounsaturated fatty acids; and ∑*n*-3 HUFAs, total *n*-3 highly unsaturated fatty acids.

The two clusters clearly classified the FA profiles of the whole-body red sea bream (cluster 1 [the Con, MM20, and MM40 diets] and cluster 2 [the MM60, MM80, and MM100 diets]) (Figure 6A). The 60% variation in the PCA model was explained by the first two principal components (PC1:46% and PC2:14%). The ∑*n*-3 HUFA, docosahexaenoic acid (DHA, C22:6*n*-3), eicosapentaenoic acid (EPA, C20:5*n*-3), myristic acid (C14:0), tetracosenoic acid (C24:1*n*-9), palmitoleic acid (C16:1*n*-7), myristoleic acid (C14:1*n*-5), docosadienoic acid (C22:2*n*-6), and arachidonic acid (C20:4*n*-6) were identified as the most significant variables in PC1 (Figure 6B). The eicosadienoic acid (C20:2*n*-6) and eicosenoic acid (C20:1*n*-9) were identified as the most significant variables in PC2. Furthermore, ∑*n*-3 HUFA, DHA, EPA, arachidonic acid, docosadienoic acid, *ω*-3 eicosatrienoic acid (C20:3*n*-3), *ω*-6 eicosatrienoic acid (C20:3*n*-6), myristic acid, palmitoleic acid, myristoleic acid, pentadecenoic acid (C15:1*n*-5), and tetracosenoic acid, were strongly correlated with the FA profiles of the whole-body fish fed the Con, MM20, and MM40 diets. The ∑SFA, ∑MUFA, oleic acid (C18:1*n*-9), and palmitic acid (C16:0) were strongly correlated with the FA profiles of the whole-body fish fed the MM60, MM80, and MM100 diets.

The ∑SFA of the whole-body fish was not statistically (*p* > 0.1) altered by dietary FM substitution with MM (Table 6). However, the ∑MUFA of the whole-body fish fed the MM60, MM80, and MM100 diets was statistically (*p* < 0.0001) higher than that of fish fed the Con and MM20 diets, but not statistically (*p* > 0.05) different from that of fish fed the MM40 diet. The whole-body ∑*n*-3 HUFA of fish fed the Con diet was statistically (*p* < 0.0001) higher than that of fish fed all other feeds. In orthogonal polynomial contrast, the ∑MUFA and ∑*n*-3 HUFA of the whole-body fish revealed linear (*p* ≤ 0.001 for both) and quadratic (*p*=0.036 and *p*=0.006, respectively) relationships with dietary FM substitution levels by MM. In regression analysis, linear relationships were revealed to be the most suitable model between dietary elevated MM substitution for FM (X) and the whole-body ∑MUFA (*Y* = 0.013629*X* + 39.8786, *R*^2^ = 0.7808, *p* < 0.0001) and ∑*n*-3 HUFA (*Y* = –0.024529*X* + 6.4414, *R*^2^ = 0.9664, *p* < 0.0001) of fish, respectively.

## 4. Discussion

Terrestrial animal proteins have been widely adopted as a FM substitute in diets of diverse fish species [8, 19]. In particular, high-quality MM has been positively evaluated for its potential use as a substitute for FM in fish feeds due to its high protein and low ash content [7] and comparable or superior digestibility compared to FM [25]. Comparable weight gain and SGR of red sea bream fed the MM40 diet to fish fed the Con diet in the current experiment indicated that up to 40% of FM in a 55% FM-based diet could be replaced with high-quality MM without significantly causing undesirable impact on growth performance of red sea bream. However, the MM60, MM80, and MM100 diets led to inferior growth performance of fish compared to the Con diet, suggesting that high (≥60%) FM replacement by MM in the red sea bream feed was not desirable. Likewise, replacement of FM up to 40% with high-quality MM in the 65% FM-based diet [7], and up to 60% with high-quality MM in the 80% FM-basal diet with AA supplementation [21] have revealed no compromising growth performance of olive flounder. However, FM up to 30% could be replaced by porcine MM in a 30% FM-basal diet without deteriorating the growth of golden pompano [27]. In addition, MM (CP: 74.4%) could replace FM up to 29.1% without compromising the growth performance of rockfish when fish were provided with a 55% FM-based diet or diets substituting graded (14.5%–58.2%) levels of FM by MM [22]. These outcomes might imply that substitutability of FM by MM in fish diets appeared to be highly influenced by MM quality (CP content) and/or supplementation of EAA.

Furthermore, dietary increased FM replacement with high-quality MM led to a linear decrease in the growth performance of red sea bream, which might be due to increased carbohydrate content in the experimental diets (Table 1). Carnivorous fish including red sea bream have a limited ability to utilize dietary carbohydrates [44, 45] due to their low capacity for glucose metabolism [44]. In addition, dietary carbohydrate must be digested to provide energy for fish [46], but excessive glucose can impair energy metabolism in fish [47], making growth less efficient. Fish size is another factor that affects glucose tolerance, with larger (4.5 g) tilapia having better glucose tolerance than smaller (0.5 g) tilapia [48]. Therefore, the high carbohydrate content in the experimental diets might have negatively affected the energy metabolism, with the small size of the experimental fish exacerbating this effect, resulting in reduced growth.

Fifty percent FM (30% FM protein) could be substitutable by MM in the 60% FM-based feed of fingerling (mean initial weight of 50.2 g) red sea bream in Gunathilaka et al.'s [20] study, whereas MM could substitute FM up to 40% (22% FM protein) in the 55% FM-basal feed of juvenile (mean initial weight of 7.9 g) red sea bream in the current study. This discrepancy in substitutability of FM by MM in the red sea bream diets might be attributed to the size (age) of fish, with smaller fish being more sensitive to dietary FM substitution with a replacer. Likewise, Lee, Azarm, and Chang [35] proved that fermented soybean meal could substitute FM up to 10% and 40% in the juvenile and grower rockfish feeds, respectively, without negative effects on growth performance and feed consumption when juvenile and grower rockfish were supplied with a 58% FM-basal feed or feeds replacing 10%–40% of FM by fermented soybean meal for 8 weeks. Similarly, tuna by-product meal (TBM) could replace FM up to 40% in the diets of the early stage of juvenile (mean initial weight of 2.4 g) rockfish when juvenile rockfish were supplied with a 55% FM-based diet or diets substituting diverse (20%–100%) FM levels by TBM in Li and Cho's [49] study. However, TBM could replace FM up to 75% in the feeds of the late stage of juvenile (mean initial weight of 29.5 g) rockfish when fish were fed with a 64.8% FM-based feed or feeds replacing various (25%–100%) levels of FM by TBM in Kim et al.'s [50] study.

The arginine, lysine, and valine in the experimental feeds seemed to fulfill dietary demands (2.37%, 1.79%, and 0.90% of the diet, respectively) for juvenile red sea bream [41–43] in this experiment. Although all experimental diets fulfilled dietary AA demands known for red sea bream, the growth of fish fed the MM60, MM80, and MM100 diets would be notably reduced. The decrease in all EAA at high FM substitution levels with MM in this experiment, except for arginine might partially impact the growth of red sea bream adversely. This could be demonstrated by the fact of the significant linear relationships between weight gain (*Y* = – 0.145434*X* + 31.6540, *R*^2^ = 0.9253, *p* < 0.0001) and SGR of fish (*Y* = – 0.008442*X* + 2.9195, *R*^2^ = 0.9161, *p* < 0.0001) and dietary MM replacement levels for FM in regression analysis. Likewise, high replacement levels of MBM in diets led to the imbalance of EAA, resulting in worsened growth performance of large yellow croaker [19]. Moreover, histidine influences protein synthesis and muscle development in fish, and a dietary histidine deficiency or excess has been shown to negatively affect the growth of Nile tilapia (*Oreochromis niloticus*) [51]. Leucine, isoleucine, and valine deficiency in the diet also led to decreased growth performance and FE of red drum (*Sciaenops ocellatus*) [52]. Aromatic AAs, including phenylalanine and tryptophan, play an important role in protein synthesis and physiological metabolism in animals [53], and the dietary inclusion of optimal levels of phenylalanine improved the growth, feed utilization, gut micromorphology, and immunity of hybrid grouper (*Epinephelus fuscoguttatus* × *E. lanceolatus*) [54]. The growth and immune response of olive flounder were also affected by dietary threonine content [55]. Like the aforementioned studies, dietary EAAs are important for normal growth and health of fish [52], but accurately assessing how a deficiency in individual EAA negatively influences the growth performance of red sea bream remains challenging, as the requirements for most of the EAA in their diet, except for arginine, lysine, and valine are still unknown.

Higher feed consumption of fish fed the Con, MM20, and MM40 diets compared to fish fed the MM60, MM80, and MM100 diets in this experiment indicated that MM could replace FM up to 40% without significantly deteriorating feed consumption. Feed consumption of red sea bream linearly decreased with elevated MM substitution levels for FM in regression analysis in this experiment, reflecting a reduced growth. Likewise, feed consumption of rockfish linearly decreased, and eventually lowered the growth performance with dietary elevated TBM substitution for FM [49]. Similarly, dietary elevated FM substitution levels by PBM lowered feed consumption of gilthead seabream (*Sparus aurata*), and eventually deteriorated weight gain and SGR when juvenile gilthead seabream were supplied with a 58% FM-basal diet or one of diets substituting 50% and 100% of FM by PBM for 100 days [8]. Furthermore, Karapanagiotidis et al. [8] also proved that feed consumption of juvenile gilthead seabream fed a diet substituting 25% FM with PBM or diets substituting 25% and 50% of FM by PBM with lysine and methionine supplementation was similar to fish fed a 58% FM-based diet in the 110-day feeding trial, and eventually led to no differences in growth of gilthead seabream. Dietary increased alternative source replacement for FM in excess commonly worsened palatability, lowered feed intake, and eventually led to reduced growth of fish [3, 54].

Some AAs are commonly known as feed attractants and stimulants in fish diets [54, 55]. Furthermore, significant relationships between histidine, lysine, and threonine in the red sea bream feeds and weight gain and SGR were found [56]. In considering the results of Baek and Cho's [56] study, histidine, lysine, and threonine declined with dietary elevated MM substitution levels for FM in this experiment, which might be one of the reasons why the feed consumption of red sea bream linearly decreased with the experimental diets, leading to worsened growth performance. In addition, feed consumption of fish could be impacted by FA profiles of diets [57]. Ozyurt et al. [58] also unveiled that palmitoleic acid was abundant in keeled mullet (*Liza carinata*), which was the most preferred bait for bluefish (*Pomatomus saltatrix*) among bait fish tested (keeled and leaping mullets, and sardine). Decreased palmitoleic acid with dietary increased FM substitution with MM could be another reason why high MM replacement for FM in feeds deteriorated palatability and lowered feed consumption by red sea bream.

No discernible differences in FE, PER, and PR of fish fed the MM80 diet compared to fish fed the Con diet in the current experiment might indicate that FM up to 80% could be substitutable by MM without significantly deteriorating feed utilization. In regression analysis, however, quadratic and linear relationships between FE and PER, and PR of fish and elevated FM replacement with MM in diets were observed in this experiment. This could result from the fact that high FM replacement with MM in diets could lead to an imbalanced EAA, resulting in low protein synthesis efficiency [59]. Likewise, dietary FM substitution with MM deteriorated feed utilization of olive flounder and rockfish [22, 24]. Similarly, the lowest FE and PR were attained in large yellow croaker (*Pseudosciaena crocea*) fed a feed substituting 75% of FM with MBM when large yellow croaker were supplied with a 55% FM-basal feed or feeds substituting diverse (15%–75%) FM levels with MBM [19]. In addition, FM up to 50% and 100% could be substitutable by tuna muscle by-product powder without compromising growth and feed consumption, and FE, respectively, when olive flounder were supplied with a 58.5% FM-basal feed or one of the feeds substituting various (25%–100%) levels of FM by tuna muscle by-product powder [57].

The *n*-3 HUFA is important for marine fish, specifically carnivorous fish species for normal growth and survival [60, 61]. Increased MM substitution for FM in the experimental diets linearly decreased ∑*n*-3 HUFA including EPA and DHA, which may be one of the reasons why the high FM substitution with MM in diets led to poor growth of red sea bream in the current experiment. Likewise, the dietary low level of *n*-3 HUFA led to decreased weight gain of juvenile hybrid grouper, and growth performance and FE of black seabream (*Acanthopagrus schlegeli*) compared to the dietary optimal ∑*n*-3 HUFA [62, 63]. Moreover, an appropriate ratio of DHA to EPA (DHA/EPA) in diets improved growth performance of fish, and it was reported to be 1.40 and 2.17 based on weight gain and SGR of golden pompano [64] and large yellow croaker (*Larmichthys crocea*) [65], respectively. The DHA/EPA ranged from 1.18 in the MM100 diet to 1.73 in the Con diet in the current experiment. Although the dietary optimal DHA/EPA for red sea bream was unknown, considering the fact that DHA is twice as efficient as EPA in red sea bream [66], decreased DHA/EPA with increased dietary MM replacement for FM might contribute to deteriorated growth performance.

Over the past decades, raw fish has become widely popular worldwide [67], with consumers preferring raw fish with high CF (thick fillet). CF is used as an indicator to determine growth pattern and health condition of fish [68] and the linear decrease in CF of red sea bream agreed with growth rate in this experiment. Likewise, CF linearly decreased with decreased growth rate in turbot (*Psetta maxima*) and gilthead seabream when dietary FM was replaced by wheat gluten and hydrolyzed feather meal, respectively [69, 70]. In addition, VSI and HSI are the indicators to estimate the amount of visceral fat accumulation [71] and the metabolic activity of fish by evaluating the liver size of fish [72], respectively. No discernible differences in VSI and HSI were found among fish in this experiment, indicating that increased FM substitution by MM in the feed did not lead to any undesirable impact on visceral and metabolic status in red sea bream. Similarly, chicken waste meal (CWM) could substitute FM up to 28.6% in a 35% FM-basal feed without compromising growth of Asian seabass (*Lates calcarifer*) and the growth rate of fish was well reflected in CF, but VSI and HSI were unaffected when juvenile Asian seabass were supplied with the 35% FM-basal feed or one of feeds replacing graded (14.3%–57.1%) levels of FM by CWM for 8 weeks [73]. However, dietary FM replacement with porcine MM, MM, and MBM did not influence CF, VSI, and HSI of golden pompano, rockfish, and gilthead seabream, respectively [4, 22, 27].

Plasma parameters have been used to assess the nutritional, clinical, and physiological status of fish [74]. All plasma measurements of fish were unaffected by FM replacement by MM in feeds in the present experiment, suggesting that dietary FM substitution by MM did not deteriorate plasma parameters. Likewise, dietary MM substitution for FM did not influence plasma measurements of rockfish [22, 24]. Furthermore, the whole FM substitution by PBM in diet did not impact plasma measurements (AST, ALT, TP, ALB, urea, and creatinine) of Nile tilapia [75]. Serum SOD is recognized as one of the crucial antioxidant enzymes that protect all aerobic life from harmful oxidative damage induced by reactive oxygen species [76] and lysozyme activity is a crucial primary defense mechanism in fish against pathogens [77]. Serum parameters of fish were also unaffected by dietary treatments, indicating that dietary MM substitution for FM did not induce any negative effect on serum SOD and lysozyme activity. Similarly, serum lysozyme activity and SOD of rockfish were not affected by dietary MM substitution for FM [24]. In addition, dietary 30% FM replacement by diverse swine by-product meals (plasma, heme, and globin powders, and pork greaves meal) did not alter serum SOD and lysozyme activity of olive flounder [78]. Unlike the current study, however, serum lysozyme activity of black carp (*Mylopharyngodon piceus*) tended to decrease with elevated FM replacement with blood meal in diets [77].

The proximate composition and AA profiles of the whole-body red sea bream were not impacted by increased FM replacement by MM in feeds in the current experiment, indicating that FM substitution with MM in feeds did not induce any difference in the proximate composition and AA profiles. Likewise, the whole-body chemical composition and AA profiles of olive flounder were not altered by dietary FM replacement by diverse swine by-product meals [78]. Similarly, dietary FM replacement with porcine blood meal and PBM did not change the proximate composition of the whole-body red sea bream and Nile tilapia, respectively [5, 75]. Similarly, dietary MM substitution for FM did not change the proximate composition and AA profiles of the whole-body olive flounder, except for alanine [7]. In addition, the proximate composition of gilthead seabream was altered by dietary MBM replacement for FM, but not for the AA profiles [4]. No discernible differences in the AA profiles of fish reflected from dietary FM replacement with substitutes could be explained by the fact that proteins in the body of fish are synthesized based on genetic information in DNA, so the whole-body AA profiles are the same regardless of dietary FM replacement by alternative sources [79].

The two clusters of the FA profiles of the whole-body red sea bream were clearly separated based on the FM replacement level (cluster 1 [the Con, MM20, and MM40 diets] and cluster 2 [the MM60, MM80, and MM100 diets]). Cluster 1 was strongly correlated with ∑*n*-3 HUFA including DHA and EPA, while cluster 2 was strongly correlated with ∑SFA and ∑MUFA. These findings suggest that these FA contributed to the differentiation among the dietary treatments, possibly due to their high content in each diet.

Increased ∑MUFA, but decreased ∑*n*-3 HUFA in the whole-body red sea bream were well reflected from dietary ∑MUFA and ∑*n*-3 HUFA in this study. Similarly, dietary elevated MM replacement for FM increased ∑MUFA, but decreased ∑*n*-3 HUFA, which were well reflected in the increased ∑MUFA, but lowered ∑*n*-3 HUFA of the whole-body olive flounder [7]. Likewise, dietary elevated FM substitution with TBM decreased dietary ∑*n*-3 HUFA, which were well reflected in the decreased ∑*n*-3 HUFA of the whole-body rockfish [49]. In addition, dietary elevated FM replacement with black soldier fly larvae (*Hermetia illucens*, L.) increased ∑SFA and EPA and DHA, but decreased ∑MUFA, which were well reflected in increased ∑SFA and EPA and DHA, but decreased ∑MUFA of the whole-body Atlantic salmon (*Salmo salar*) [80]. Furthermore, increased FM replacement with raw and cooked crocodile MM in diets lowered EPA and DHA, which led to decreased EPA and DHA of the fillet of dusky kob (*Argyrosomus japonicus*) [81].

## 5. Conclusion

MM could substitute 40% FM in diets without significantly compromising the growth and feed consumption of red sea bream. However, weight gain, SGR, and feed consumption of fish tended to lower with dietary increased MM substitution for FM. Nevertheless, no discernible differences in the biological indices, except for CF, blood chemistry, proximate composition, and AA profiles of fish were found among dietary treatments.

## Figures and Tables

**Table 1:** Ingredients and chemical composition of the experimental diets (%, DM basis).

	Experimental diets					
	Con	MM20	MM40	MM60	MM80	MM100
Ingredients (%, DM)						
Fish meal (FM; anchovy meal)^a^	55.0	44.0	33.0	22.0	11.0	—
Meat meal (MM)^b^	—	9.5	19.0	28.3	37.5	47.0
Fermented soybean meal	17.0	17.0	17.0	17.0	17.0	17.0
Wheat flour	17.5	18.8	20.1	21.6	23.2	24.5
Fish oil	4.0	4.2	4.4	4.6	4.8	5.0
Soybean oil	4.0	4.0	4.0	4.0	4.0	4.0
Vitamin premix^c^	1.0	1.0	1.0	1.0	1.0	1.0
Mineral premix^d^	1.0	1.0	1.0	1.0	1.0	1.0
Choline	0.5	0.5	0.5	0.5	0.5	0.5
Nutrients (%, DM)						
Dry matter	95.7	94.8	94.6	94.6	94.9	95.1
Crude protein	51.3	51.6	51.4	51.3	51.5	51.3
Crude lipid	14.6	14.7	14.5	14.5	14.6	14.4
Ash	10.2	9.0	8.1	7.0	6.5	5.3
Carbohydrate^e^	23.9	24.7	26.0	27.2	27.4	29.0

*Note:* Con: 55% fish meal (FM)-based diet; MM20: dietary 20% FM substitution with meat meal (MM); MM40: dietary 40% FM substitution with MM; MM60: dietary 60% FM substitution with MM; MM80: dietary 80% FM substitution with MM; MM100: dietary 100% FM substitution with MM.

^a^Fish meal (FM; anchovy meal) (CP: 73.4%, CL: 10.7%, and ash: 14.0%) was imported from Chile (USD 2.17/kg FM, USD = 1300 KRW).

^b^Meat meal (MM) (CP: 83.9%, CL: 9.9%, and ash: 6.0%) was purchased from Daekyung Oil & Transportation Co., Ltd. (Busan Metropolitan City, Korea) (USD 0.88/kg MM).

^c^Vitamin premix (g/kg mix): L-ascorbic acid, 121.2; DL-*α*-tocopheryl acetate, 18.8; thiamine hydrochloride, 2.7; riboflavin, 9.1; pyridoxine hydrochloride, 1.8; niacin, 36.4; Ca-D-pantothenate, 12.7; myo-inositol, 181.8; D-biotin, 0.27; folic acid, 0.68; p-aminobenzoic acid, 18.2; menadione, 1.8; retinyl acetate, 0.73; cholecalciferol, 0.003; cyanocobalamin, 0.003.

^d^Mineral premix (g/kg mix): MgSO_4_^.^7H_2_O, 80.0; NaH_2_PO_4_^.^2H_2_O, 370.0; KCl, 130.0; ferric citrate, 40.0; ZnSO_4_^.^7H_2_O, 20.0; Ca-lactate, 356.5; CuCl, 0.2; AlCl_3_^.^6H_2_O, 0.15; KI, 0.15; Na_2_Se_2_O_3_, 0.01; MnSO_4_^.^H_2_O, 2.0; CoCl_2_^.^6H_2_O, 1.0.

^e^Carbohydrate was calculated by difference (carbohydrate = 100 – [crude protein + crude lipid + ash]).

**Table 2:** Survival (%), feed efficiency (FE), protein efficiency ratio (PER), protein retention (PR, %), condition factor (CF, g/cm^3^), viscerosomatic index (VSI, %), and hepatosomatic index (HSI, %) of red sea bream fed the experimental diets for 8 weeks.

	Experimental diets						PSEM	*p*-Value	Orthogonal polynomial contrast			Regression analysis		
	Con	MM20	MM40	MM60	MM80	MM100			Linear	Quadratic	Cubic	Model	*p*-Value	*R* ^2^
Initial weight (g/fish)	7.81	7.84	7.87	7.87	7.89	7.84	0.148	—	—	—	—	—	—	—
Final weight (g/fish)	37.77	37.26	35.50	31.33	27.24	24.31	1.250	—	—	—	—	—	—	—
Survival (%)	90.67	90.67	92.00	92.00	88.00	81.33	1.674	*p* > 0.7	0.138	0.172	0.621	NR	—	—
FE^a^	0.97^a,b^	0.98^a^	0.97^a,b^	0.95^a,b^	0.92^a,b^	0.90^b^	0.009	*p* < 0.04	0.002	0.169	0.715	*Y* = –0.00001051*X*^2^ + 0.000226*X* + 0.9763	*p* < 0.001	0.5908
PER^b^	1.84^a^	1.84^a^	1.83^a^	1.81^a^	1.76^a,b^	1.67^b^	0.018	*p* < 0.01	0.001	0.052	0.465	*Y* = –0.00002583*X*^2^ + 0.000987*X* + 1.8367	*p* < 0.0001	0.6685
PR (%)^c^	31.19^a^	30.86^a^	30.54^a^	30.17^a,b^	29.60^a,b^	28.07^b^	0.295	*p* < 0.006	0.000	0.124	0.402	*Y* = –0.028218*X* + 31.4833	*p* < 0.0001	0.6270
CF (g/cm^3^)^d^	1.92^a^	1.88^a,b^	1.87^a,b^	1.77^a,b,c^	1.69^b,c^	1.58^c^	0.032	*p* < 0.001	0.000	0.178	0.900	*Y* = –0.003384*X* + 1.9533	*p* < 0.0001	0.7451
VSI (%)^e^	8.73	8.70	8.61	8.64	8.64	8.63	0.077	*p* > 0.9	0.724	0.830	0.941	NR	—	—
HSI (%)^f^	2.81	2.82	2.74	2.77	2.70	2.71	0.038	*p* > 0.9	0.368	0.958	0.873	NR	—	—

*Note:* Con: 55% fish meal (FM)–based diet; MM20: dietary 20% FM substitution with meat meal (MM); MM40: dietary 40% FM substitution with MM; MM60: dietary 60% FM substitution with MM; MM80: dietary 80% FM substitution with MM; MM100: dietary 100% FM substitution with MM; PSEM: pooled standard error of means; *R*^2^: *R*-squared value. Values (means of triplicates) in the same row sharing the same superscript letter are not statistically different (*p*  > 0.05).

Abbreviations: CF, condition factor; FE, feed efficiency; HSI, hepatosomatic index; NR, no relationship; PER, protein efficiency ratio; PR, protein retention; VSI, viscerosomatic index.

^a^FE = (Total final weight of fish [g] − total initial weight of fish [g] + total weight of dead fish [g])/total feed consumption (g).

^b^PER = Weight gain of fish (g)/protein consumption (g).

^c^PR (%) = Protein gain of fish (g) × 100/protein consumption (g).

^d^CF (g/cm^3^) = Body weight of fish (g) × 100/total length of fish (cm)^3^.

^e^VSI (%) = Viscera weight of fish (g) × 100/body weight of fish (g).

^f^HSI (%) = Liver weight of fish (g) × 100/body weight of fish (g).

**Table 3:** Plasma and serum parameters of red sea bream fed the experimental diets for 8 weeks.

	Experimental diets						PSEM	*p*-Value	Orthogonal polynomial contrast			Regression analysis		
	Con	MM20	MM40	MM60	MM80	MM100			Linear	Quadratic	Cubic	Model	*p*-Value	*R* ^2^
Plasma parameters														
AST (U/L)	49.3	50.2	49.8	51.1	50.8	51.3	0.51	*p* > 0.8	0.288	0.926	0.955	NR	—	—
ALT (U/L)	7.4	7.4	7.4	7.7	7.4	7.3	0.10	*p* > 0.9	0.861	0.641	0.687	NR	—	—
ALP (U/L)	178.2	173.9	169.2	172.9	179.9	173.2	1.99	*p* > 0.7	0.939	0.483	0.269	NR	—	—
T-BIL (mg/dL)	0.3	0.3	0.4	0.3	0.4	0.4	0.03	*p* > 0.9	0.352	0.964	0.733	NR	—	—
T-CHO (mg/dL)	257.2	259.3	271.1	261.0	260.7	258.7	4.07	*p* > 0.9	0.992	0.522	0.807	NR	—	—
TG (mg/dL)	407.3	415.7	416.6	371.6	400.9	412.4	7.09	*p* > 0.4	0.671	0.436	0.214	NR	—	—
TP (g/dL)	5.0	4.7	5.0	5.0	4.9	4.9	0.06	*p* > 0.8	0.981	0.762	0.617	NR	—	—
ALB (g/dL)	1.0	1.0	1.2	1.1	1.1	1.0	0.03	*p* > 0.5	0.750	0.138	0.894	NR	—	—
Serum parameters														
SOD (ng/mL)	3.9	3.8	3.8	4.1	3.9	4.2	0.12	*p* > 0.9	0.669	0.551	0.922	NR	—	—
Lysozyme activity (U/mL)	50.1	52.2	44.0	50.3	50.7	55.1	2.66	*p* > 0.9	0.514	0.659	0.880	NR	—	—

*Note:* Con: 55% fish meal (FM)–based diet; MM20: dietary 20% FM substitution with meat meal (MM); MM40: dietary 40% FM substitution with MM; MM60: dietary 60% FM substitution with MM; MM80: dietary 80% FM substitution with MM; MM100: dietary 100% FM substitution with MM; PSEM: pooled standard error of means; *R*^2^: *R*-squared value; NR: no relationship. Values (means of triplicates) in the same row sharing the same superscript letter are not statistically different (*p*  > 0.05).

Abbreviations: ALB, albumin; ALP, alkaline phosphatase; ALT, alanine aminotransferase; AST, aspartate aminotransferase; SOD, superoxide dismutase; T-BIL, total bilirubin; T-CHO, total cholesterol; TGs, triglycerides; TP, total protein.

**Table 4:** The whole-body proximate composition (% of wet weight) of red sea bream fed the experimental diets for 8 weeks.

	Experimental diets						PSEM	*p*-Value	Orthogonal polynomial contrast			Regression analysis		
	Con	MM20	MM40	MM60	MM80	MM100			Linear	Quadratic	Cubic	Model	*p*-Value	*R* ^2^
Moisture	68.9	69.2	68.9	69.2	68.5	68.5	0.05	*p* > 0.08	0.030	0.147	0.411	NR	—	—
Crude protein	16.8	16.7	16.6	16.6	16.4	16.7	0.04	*p* > 0.05	0.041	0.095	0.106	NR	—	—
Crude lipid	8.7	8.6	8.7	8.5	8.6	8.3	0.05	*p* > 0.2	0.029	0.348	0.411	NR	—	—
Ash	4.4	4.3	4.4	4.4	4.4	4.7	0.05	*p* > 0.09	0.032	0.044	0.979	NR	—	—

*Note:* Con: 55% fish meal (FM)–based diet; MM20: dietary 20% FM substitution with meat meal (MM); MM40: dietary 40% FM substitution with MM; MM60: dietary 60% FM substitution with MM; MM80: dietary 80% FM substitution with MM; MM100: dietary 100% FM substitution with MM; *R*^2^: *R*-squared value.

Abbreviations: NR, no relationship; PSEMs, pooled standard error of means.

**Table 5:** The whole-body amino acid (% of wet weight) profiles of red sea bream fed the experimental diets for 8 weeks.

	Experimental diets						PSEM	*p*-Value	Orthogonal polynomial contrast			Regression analysis		
	Con	MM20	MM40	MM60	MM80	MM100			Linear	Quadratic	Cubic	Model	*p*-Value	*R* ^2^
Essential amino acids (EAAs) (%)														
Arginine	1.00	1.01	1.03	1.04	1.05	1.03	0.008	*p* > 0.1	0.008	0.770	0.776	NR	—	—
Histidine	0.36	0.34	0.35	0.34	0.33	0.33	0.006	*p* > 0.5	0.067	0.929	0.928	NR	—	—
Isoleucine	0.57	0.57	0.55	0.53	0.54	0.52	0.008	*p* > 0.3	0.035	0.881	0.592	NR	—	—
Leucine	1.14	1.12	1.11	1.12	1.08	1.10	0.008	*p* > 0.1	0.005	0.966	0.749	NR	—	—
Lysine	1.28	1.27	1.27	1.25	1.25	1.24	0.007	*p* > 0.4	0.049	0.789	0.833	NR	—	—
Phenylalanine	0.61	0.61	0.60	0.59	0.58	0.58	0.006	*p* > 0.6	0.101	0.843	0.730	NR	—	—
Threonine	0.72	0.72	0.70	0.71	0.71	0.70	0.006	*p* > 0.8	0.227	0.750	0.756	NR	—	—
Tryptophan	0.12	0.12	0.11	0.11	0.10	0.09	0.005	*p* > 0.3	0.036	0.621	0.555	NR	—	—
Valine	0.66	0.65	0.63	0.64	0.63	0.63	0.006	*p* > 0.8	0.211	0.663	0.952	NR	—	—
Non-essential amino acids (NEAAs) (%)														
Alanine	1.10	1.12	1.12	1.14	1.13	1.13	0.006	*p* > 0.7	0.178	0.540	0.717	NR	—	—
Aspartic acid	1.50	1.49	1.49	1.48	1.46	1.46	0.007	*p* > 0.5	0.074	0.743	0.779	NR	—	—
Glutamic acid	2.14	2.18	2.17	2.19	2.18	2.20	0.008	*p* > 0.2	0.019	0.819	0.677	NR	—	—
Glycine	1.31	1.32	1.36	1.35	1.36	1.38	0.009	*p* > 0.1	0.008	0.971	0.999	NR	—	—
Proline	0.78	0.80	0.81	0.83	0.82	0.84	0.007	*p* > 0.3	0.026	0.867	0.733	NR	—	—
Serine	0.72	0.75	0.74	0.75	0.76	0.76	0.007	*p* > 0.4	0.042	0.666	0.701	NR	—	—
Tyrosine	0.44	0.44	0.44	0.43	0.43	0.42	0.005	*p* > 0.9	0.411	0.587	0.833	NR	—	—

*Note:* Con: 55% fish meal (FM)–based diet; MM20: dietary 20% FM substitution with meat meal (MM); MM40: dietary 40% FM substitution with MM; MM60: dietary 60% FM substitution with MM; MM80: dietary 80% FM substitution with MM; MM100: dietary 100% FM substitution with MM; *R*^2^: *R*-squared value.

Abbreviations: NR, no relationship; PSEMs, pooled standard error of means.

**Table 6:** The whole-body fatty acid (% of total fatty acids) profiles of red sea bream fed the experimental diet for 8 weeks.

	Experimental diets						PSEM	*p*-Value	Orthogonal polynomial contrast			Regression analysis		
	Con	MM20	MM40	MM60	MM80	MM100			Linear	Quadratic	Cubic	Model	*p*-Value	*R* ^2^
C14:0	1.63^a^	1.62^a^	1.50^a,b^	1.44^b^	1.40^b^	1.39^b^	0.027	*p* < 0.001	0.000	0.378	0.267	*Y* = – 0.00279029*X* + 1.6373	*p* < 0.0001	0.7477
C16:0	15.90^a^	15.97^a,b^	16.15^b,c^	16.23^b,c^	16.40^c^	16.61^c^	0.065	*p* < 0.0001	0.000	0.361	0.819	*Y* = 0.007081*X* + 15.8565	*p* < 0.0001	0.8183
C18:0	7.17	7.21	7.21	7.26	7.27	7.27	0.019	*p* > 0.6	0.101	0.732	0.854	NR	—	—
C20:0	0.21	0.21	0.21	0.22	0.22	0.23	0.007	*p* > 0.9	0.384	0.854	0.940	NR	—	—
C22:0	0.50	0.54	0.54	0.55	0.57	0.58	0.013	*p* > 0.5	0.086	0.787	0.633	NR	—	—
C24:0	0.52	0.50	0.49	0.46	0.46	0.43	0.012	*p* > 0.3	0.033	0.845	0.855	NR	—	—
∑SFA^a^	25.93	26.05	26.10	26.17	26.32	26.51	0.068	*p* > 0.1	0.009	0.561	0.697	NR	—	—
C14:1*n*-5	0.06	0.05	0.05	0.04	0.03	0.03	0.004	*p* > 0.4	0.046	0.894	0.821	NR	—	—
C15:1*n*-5	0.06	0.04	0.04	0.04	0.03	0.03	0.005	*p* > 0.6	0.150	0.695	0.518	NR	—	—
C16:1*n*-7	3.45^a^	3.40^a^	3.32^a,b^	3.12^b,c^	2.93^c^	2.69^d^	0.067	*p* < 0.0001	0.000	0.004	0.629	*Y* = –0.007724*X* + 3.5395	*p* < 0.0001	0.9044
C17:1*n*-7	0.42	0.40	0.40	0.39	0.38	0.38	0.011	*p* > 0.8	0.229	0.806	0.920	NR	—	—
C18:1*n*-9	32.55^f^	33.14^e^	33.90^d^	34.43^c^	34.98^b^	35.36^a^	0.240	*p* < 0.0001	0.000	0.016	0.368	*Y* = 0.028729*X* + 32.6230	*p* < 0.0001	0.9800
C20:1*n*-9	1.35	1.33	1.33	1.32	1.31	1.31	0.012	*p* > 0.9	0.282	0.889	0.936	NR	—	—
C22:1*n*-9	0.53	0.51	0.50	0.50	0.49	0.49	0.011	*p* > 0.9	0.331	0.718	0.938	NR	—	—
C24:1*n*-9	1.31^a^	1.21^a,b^	1.13^b,c^	0.97^c,d^	0.84^d,e^	0.78^e^	0.048	*p* < 0.0001	0.000	0.977	0.274	*Y* = –0.00559024*X* + 1.3195	*p* < 0.0001	0.9177
∑MUFA^b^	39.73^c^	40.08^b,c^	40.68^a,b^	40.81^a^	40.99^a^	41.07^a^	0.128	*p* < 0.0001	0.000	0.036	0.919	*Y* = 0.013629*X* + 39.8786	*p* < 0.0001	0.7808
C18:2*n*-6	22.18	22.35	22.57	22.67	22.80	23.23	0.134	*p* > 0.2	0.023	0.747	0.692	NR	—	—
C18:3*n*-3	2.61	2.60	2.58	2.57	2.57	2.55	0.017	*p* > 0.9	0.285	0.976	0.975	NR	—	—
C18:3*n*-6	0.63	0.63	0.62	0.61	0.61	0.60	0.010	*p* > 0.9	0.334	0.970	0.980	NR	—	—
C20:2*n*-6	0.38	0.38	0.37	0.36	0.34	0.34	0.010	*p* > 0.6	0.116	0.786	0.704	NR	—	—
C20:3*n*-3	0.02	0.02	0.02	0.01	0.01	0.01	0.002	*p* > 0.6	0.150	0.948	0.403	NR	—	—
C20:3*n*-6	0.02	0.02	0.02	0.01	0.01	0.01	0.002	*p* > 0.6	0.201	0.763	0.230	NR	—	—
C20:4*n*-6	0.73	0.70	0.67	0.66	0.62	0.60	0.014	*p* > 0.06	0.003	0.920	0.986	NR	—	—
C20:5*n*-3	2.47^a^	2.30^a,b^	2.16^b^	1.80^c^	1.68^c,d^	1.48^d^	0.087	*p* < 0.0001	0.000	0.820	0.237	*Y* = –0.010200*X* + 2.4922	*p* < 0.0001	0.9470
C22:2*n*-6	0.41	0.38	0.36	0.33	0.31	0.31	0.013	*p* > 0.1	0.007	0.484	0.644	NR	—	—
C22:6*n*-3	4.08^a^	3.65^b^	3.19^c^	2.95^c,d^	2.75^d^	2.68^d^	0.124	*p* < 0.0001	0.000	0.001	0.835	*Y* = –0.014229*X* + 3.9270	*p* < 0.0001	0.9023
∑*n*-3 HUFA^c^	6.57^a^	5.97^b^	5.37^c^	4.77^d^	4.45^e^	4.17^e^	0.207	*p* < 0.0001	0.000	0.006	0.228	*Y* = –0.024529*X* + 6.4414	*p* < 0.0001	0.9664
Unknown	0.81	0.83	0.67	1.05	0.98	0.60	0.089	—	—	—	—	—	—	—

*Note:* Con: 55% fish meal (FM)–based diet; MM20: dietary 20% FM substitution with meat meal (MM); MM40: dietary 40% FM substitution with MM; MM60: dietary 60% FM substitution with MM; MM80: dietary 80% FM substitution with MM; MM100: dietary 100% FM substitution with MM; *R*^2^: *R*-squared value. Values (means of triplicates) in the same row sharing the same superscript letter are not statistically different (*p* > 0.5).

Abbreviations: NR, no relationship; PSEMs, pooled standard error of means.

^a^∑SFA: total saturated fatty acids.

^b^∑MUFA: total monounsaturated fatty acids.

^c^∑*n*-3 HUFA: total *n*-3 highly unsaturated fatty acids.

## Data Availability

The data that support the findings of this study are available from the corresponding author upon reasonable request.

## Supporting Information

Supporting Information Table S1. Amino acid (% of the diet) profiles of the main proteins and the experimental diets. Table S2. Fatty acid (% of total fatty acid) profiles of the main proteins and the experimental diets.
