# Supplementary material for: Effect of Partial or Complete Substitution of Fish Meal by Meat Meal in the Feed of Red Sea Bream (Pagrus major) on the Growth Performance and Feed Utilization
Source: Aquac Nutr. 2025 Mar 27;2025:9589317. doi: 10.1155/anu/9589317 (PMC11968162; doi:10.1155/anu/9589317)
Supplement: Supporting Information — Table S1. Amino acid (% of the diet) profiles of the main proteins and the experimental diets. Table S2. Fatty acid (% of total fatty acid) profiles of the main proteins and the experimental diets. [file 9589317.f1.docx]

|  | Main proteins | |  |  |  | Experimental diets | | | | | |
| --- | --- | --- | --- | --- | --- | --- | --- | --- | --- | --- | --- |
|  | FM | MM |  | Requirement |  | Con | MM20 | MM40 | MM60 | MM80 | MM100 |
| *Essential amino acids (EAA) (%)* | | | | | | | | | | | |
| Arginine | 3.87 | 5.62 |  | 2.37^a^ |  | 2.58 | 2.71 | 2.85 | 2.99 | 3.08 | 3.24 |
| Histidine | 1.89 | 1.48 |  |  |  | 1.38 | 1.31 | 1.23 | 1.17 | 1.08 | 1.04 |
| Isoleucine | 2.89 | 1.88 |  |  |  | 1.70 | 1.62 | 1.49 | 1.32 | 1.15 | 1.01 |
| Leucine | 5.03 | 4.12 |  |  |  | 3.82 | 3.69 | 3.32 | 3.05 | 2.85 | 2.67 |
| Lysine | 5.36 | 4.20 |  | 1.79^b^ |  | 3.68 | 3.53 | 3.41 | 3.20 | 3.08 | 2.95 |
| Phenylalanine | 2.64 | 2.42 |  |  |  | 2.12 | 1.84 | 1.78 | 1.64 | 1.54 | 1.51 |
| Threonine | 2.95 | 2.38 |  |  |  | 2.30 | 2.15 | 2.08 | 1.92 | 1.86 | 1.67 |
| Tryptophan | 0.69 | 0.33 |  |  |  | 0.36 | 0.34 | 0.31 | 0.30 | 0.26 | 0.24 |
| Valine | 3.34 | 2.76 |  | 0.90^c^ |  | 2.30 | 1.91 | 1.85 | 1.66 | 1.41 | 1.28 |
| ∑EAA^d^ | 28.66 | 25.19 |  |  |  | 20.24 | 19.10 | 18.32 | 17.25 | 16.31 | 15.61 |
| *Non-essential amino acids (NEAA) (%)* | | | | | | | | | | | |
| Alanine | 4.16 | 6.21 |  |  |  | 2.76 | 2.88 | 3.01 | 3.13 | 3.26 | 3.35 |
| Aspartic acid | 6.07 | 5.58 |  |  |  | 4.95 | 4.77 | 4.66 | 4.34 | 4.01 | 3.83 |
| Glutamic acid | 8.34 | 9.36 |  |  |  | 6.34 | 6.36 | 6.41 | 6.43 | 6.47 | 6.51 |
| Glycine | 3.76 | 12.37 |  |  |  | 2.94 | 3.48 | 4.05 | 4.85 | 5.03 | 5.69 |
| Proline | 2.74 | 7.75 |  |  |  | 2.16 | 2.53 | 2.73 | 3.68 | 4.03 | 4.37 |
| Serine | 2.58 | 2.98 |  |  |  | 1.72 | 1.73 | 1.75 | 1.80 | 1.88 | 1.95 |
| Tyrosine | 1.82 | 1.24 |  |  |  | 1.39 | 1.25 | 1.19 | 1.04 | 1.01 | 0.99 |
| ∑NEAA^e^ | 29.47 | 45.49 |  |  |  | 22.26 | 23.00 | 23.80 | 25.27 | 25.69 | 26.69 |

TABLE S1: Amino acid (% of the diet) profiles of the main proteins and the experimental diets.

FM: fish meal; MM: meat meal; Con: 55% FM-based diet; MM20: dietary 20% FM substitution with MM; MM40: dietary 40% FM substitution with MM; MM60: dietary 60% FM substitution with MM; MM80: dietary 80% FM substitution with MM; MM100: dietary 100% FM substitution with MM.

^a^ Arginine, ^b^ lysine, and ^c^ valine requirements were obtained from Rahimnejad and Lee (2014), Forster and Ogata (1998), and Rahimnejad and Lee (2013)’s studies, respectively.

^d^ ∑EAA: total essential amino acids.

^e^ ∑NEAA: total non-essential amino acids.

|  | Main proteins | |  | Experimental diets | | | | | |
| --- | --- | --- | --- | --- | --- | --- | --- | --- | --- |
|  | FM | MM |  | Con | MM20 | MM40 | MM60 | MM80 | MM100 |
| C14:0 | 4.20 | 2.12 |  | 2.49 | 1.86 | 1.71 | 1.58 | 1.43 | 1.32 |
| C16:0 | 22.22 | 29.70 |  | 15.72 | 16.08 | 16.60 | 17.20 | 17.43 | 17.81 |
| C18:0 | 8.05 | 14.27 |  | 4.77 | 5.14 | 5.54 | 5.86 | 6.18 | 6.60 |
| C20:0 | 0.10 | 0.20 |  | 0.76 | 0.78 | 0.79 | 0.83 | 0.85 | 0.86 |
| C22:0 | 0.30 | 1.61 |  | 0.34 | 0.44 | 0.52 | 0.54 | 0.63 | 0.66 |
| C24:0 | 0.68 |  |  | 0.48 | 0.41 | 0.35 | 0.30 | 0.24 | 0.17 |
| ∑SFA^a^ | 35.55 | 47.90 |  | 24.56 | 24.71 | 25.51 | 26.31 | 26.76 | 27.42 |
| C14:1n-5 | 0.23 | 0.10 |  | 0.10 | 0.08 | 0.07 | 0.06 | 0.05 | 0.03 |
| C15:1n-5 | 0.15 | 0.03 |  | 0.10 | 0.10 | 0.08 | 0.06 | 0.05 | 0.03 |
| C16:1n-7 | 5.47 | 3.00 |  | 3.14 | 2.72 | 2.37 | 2.24 | 2.09 | 1.95 |
| C17:1n-7 | 0.78 | 0.42 |  | 0.38 | 0.36 | 0.34 | 0.31 | 0.29 | 0.26 |
| C18:1n-9 | 23.30 | 43.54 |  | 30.22 | 31.94 | 33.01 | 34.09 | 35.11 | 36.15 |
| C20:1n-9 | 1.01 | 0.87 |  | 1.01 | 1.00 | 0.99 | 0.97 | 0.95 | 0.91 |
| C22:1n-9 | 0.19 | 0.04 |  | 0.38 | 0.30 | 0.24 | 0.16 | 0.12 | 0.10 |
| C24:1n-9 | 2.69 | 0.03 |  | 1.08 | 0.94 | 0.81 | 0.65 | 0.50 | 0.36 |
| ∑MUFA^b^ | 33.82 | 48.03 |  | 36.41 | 37.44 | 37.91 | 38.54 | 39.16 | 39.79 |
| C18:2n-6 | 1.89 | 2.00 |  | 23.10 | 23.47 | 23.68 | 24.01 | 24.39 | 24.81 |
| C18:3n-3 | 0.70 | 0.02 |  | 3.74 | 3.43 | 3.27 | 3.21 | 3.07 | 2.88 |
| C18:3n-6 | 0.30 | 0.05 |  | 0.39 | 0.35 | 0.33 | 0.30 | 0.28 | 0.24 |
| C20:2n-6 | 0.07 |  |  | 0.07 | 0.07 | 0.06 | 0.06 | 0.05 | 0.03 |
| C20:3n-3 | 0.17 | 0.03 |  | 0.02 | 0.02 | 0.01 | 0.01 |  |  |
| C20:3n-6 | 0.08 | 0.04 |  | 0.02 | 0.02 | 0.02 | 0.01 | 0.01 |  |
| C20:4n-6 | 2.44 | 0.22 |  | 0.73 | 0.70 | 0.64 | 0.55 | 0.49 | 0.37 |
| C20:5n-3 (EPA) | 7.06 | 0.10 |  | 2.82 | 2.62 | 2.39 | 2.13 | 1.91 | 1.73 |
| C22:2n-6 | 0.60 | 0.03 |  | 0.39 | 0.35 | 0.30 | 0.29 | 0.24 | 0.21 |
| C22:6n-3 (DHA) | 14.71 | 0.02 |  | 4.87 | 4.20 | 3.44 | 2.75 | 2.31 | 2.04 |
| DHA/EPA | 2.08 | 0.20 |  | 1.73 | 1.60 | 1.44 | 1.29 | 1.21 | 1.18 |
| ∑n-3 HUFA^c^ | 21.94 | 0.15 |  | 7.71 | 6.84 | 5.84 | 4.89 | 4.22 | 3.77 |
| Unknown | 2.61 | 1.56 |  | 2.88 | 2.62 | 2.44 | 1.83 | 1.33 | 0.48 |

TABLE S2: Fatty acid (% of total fatty acid) profiles of the main proteins and the experimental diets.

FM: fish meal; MM: meat meal; Con: 55% FM-based diet; MM20: dietary 20% FM substitution with MM; MM40: dietary 40% FM substitution with MM; MM60: dietary 60% FM substitution with MM; MM80: dietary 80% FM substitution with MM; MM100: dietary 100% FM substitution with MM; EPA: eicosapentaenoic acid; DHA: docosahexaenoic acid; DHA/EPA: the ratio of DHA to EPA.

^a^ ∑SFA: total saturated fatty acids.

^b^ ∑MUFA: total monounsaturated fatty acids.

^c^ ∑n-3 HUFA: total n-3 highly unsaturated fatty acids.
